# Identification of Uncultured Bacterial Species from Firmicutes, Bacteroidetes and *CANDIDATUS* Saccharibacteria as Candidate Cellulose Utilizers from the Rumen of Beef Cows

**DOI:** 10.3390/microorganisms6010017

**Published:** 2018-02-24

**Authors:** Lee James Opdahl, Michael G. Gonda, Benoit St-Pierre

**Affiliations:** Department of Animal Science, South Dakota State University, Animal Science Complex, Box 2170, Brookings, SD 57007, USA; Lee.Opdahl@wsu.edu (L.J.O.); Michael.Gonda@sdstate.edu (M.G.G.)

**Keywords:** rumen, bacteria, cellulose, microbiome, cattle, Firmicutes, Bacteroidetes, Saccharibacteria

## Abstract

The ability of ruminants to utilize cellulosic biomass is a result of the metabolic activities of symbiotic microbial communities that reside in the rumen. To gain further insight into this complex microbial ecosystem, a selection-based batch culturing approach was used to identify candidate cellulose-utilizing bacterial consortia. Prior to culturing with cellulose, rumen contents sampled from three beef cows maintained on a forage diet shared 252 Operational Taxonomic Units (OTUs), accounting for 41.6–50.0% of bacterial 16S rRNA gene sequences in their respective samples. Despite this high level of overlap, only one OTU was enriched in cellulose-supplemented cultures from all rumen samples. Otherwise, each set of replicate cellulose supplemented cultures originating from a sampled rumen environment was found to have a distinct bacterial composition. Two of the seven most enriched OTUs were closely matched to well-established rumen cellulose utilizers (*Ruminococcus* *flavefaciens* and *Fibrobacter* *succinogenes*), while the others did not show high nucleotide sequence identity to currently defined bacterial species. The latter were affiliated to *Prevotella* (1 OTU), Ruminococcaceae (3 OTUs), and the candidate phylum Saccharibacteria (1 OTU), respectively. While further investigations will be necessary to elucidate the metabolic function(s) of each enriched OTU, these results together further support cellulose utilization as a ruminal metabolic trait shared across vast phylogenetic distances, and that the rumen is an environment conducive to the selection of a broad range of microbial adaptations for the digestion of plant structural polysaccharides.

## 1. Introduction

As the main component of plant cell walls and sink for photosynthesis products, cellulose represents one of the most abundant organic polymers and reserve of monosaccharides on earth. Consequently, herbivory has successfully evolved in a number of different animal groups to take advantage of this abundant source of energy. However, since genes encoding cellulose-metabolizing enzymes have yet to be identified in the genomes of mammals or other animals, herbivores typically rely on gastrointestinal microbial symbionts for effective digestion and fermentation of plant-cell wall polysaccharides into metabolites that can be absorbed and assimilated [1]. 

Because cellulose and other components of plant fibers represent substrates that are difficult to digest even for microorganisms that can metabolize them, two main strategies for mammalian herbivory have successfully evolved to maximize their digestion efficiency. In hindgut fermenters, the distal segments of the gastrointestinal tract are the most developed, resulting in increased intestinal volume to favor retention time and maintenance of high microbial numbers [2]. In contrast, ruminants have developed a compartmentalized stomach, in which the largest segment, the rumen, hosts the microbial symbionts responsible for digesting the feed that they consume [3]. Ruminants represent a highly successful biological group that has not only thrived across a wide range of habitats, but also played a central role throughout human history [4]. Even to this day, the ability of domesticated ruminants to transform inedible plant biomass into protein-rich products that can be consumed by humans is expected to greatly contribute in meeting the demands of a rapidly growing and urbanizing global population [5]. 

Rumen microbial communities are complex ecosystems that form through the association of a diverse array of bacterial, archaeal, protozoal, and fungal species. Due to their anaerobic lifestyle, rumen microorganisms tend to have specialized metabolic functions, thus needing to form co-dependent trophic relationships with other members of their community to maximize their efficiency [6]. Since bacteria are found at the highest cell densities and represent the most genetically diverse group with the highest metabolic potential, they have been the focus of most investigations on rumen microbial function [1]. These have revealed that bacterial community composition tends to vary even amongst individuals of the same breed under the same diets [7]. While a number of rumen isolates, including *Prevotella ruminicola*, *Ruminococcus albus*, and *Fibrobacter succinogenes*, have been well studied and characterized, as a group they typically only represent a limited percentage of rumen cellulose-utilizing bacterial species in a given host [8,9]. In support of these observations, a number of rumen metagenomics studies have reported an abundance of candidate carbohydrate-utilizing genes that could not be assigned to known species, suggesting that a large number of uncharacterized bacteria involved in cellulose breakdown have yet to be isolated [10,11,12,13,14]. 

Given the vast diversity in rumen bacterial community assembly reported to date and the high level of functional redundancy anticipated amongst microorganisms in this ecosystem [15], we aimed to compare the composition of rumen bacteria among individuals of a beef cattle herd maintained on a forage diet, and identify previously uncharacterized utilizers of cellulose. Amongst the seven Operational Taxonomic Units (OTUs) most enriched in response to cellulose, five had not been previously isolated in culture. Intriguingly, while a core microbiome of common rumen OTUs was identified across the sampled animals, only a very limited number of OTUs were shared among cellulose-enriched consortia that were selected in vitro. 

## 2. Materials and Methods 

### 2.1. Sample Collection and In Vitro Rumen Culture Experiments

All animal procedures were approved by the Institutional Animal Care and Use Committee (IACUC) at South Dakota State University. Fistulated Angus beef cows maintained at the South Dakota State University Cow/Calf Research and Teaching Unit were used as the source of rumen fluid for batch culturing experiments. The diet of these animals consisted of pasture hay or haylage during the period when rumen fluid was collected (December 2015 to October 2016). For each batch culture experiment, the following procedure was followed. Fresh rumen contents, collected from a single animal, was squeezed by hand to obtain 12 L of the liquid fraction, which was stored in insulated containers. Within one hour of collection from the host animal, batch cultures were setup by combining and mixing the rumen fluid from different containers, which was then distributed amongst five bioreactors (Chemglass). Volumes of approximately 2.2 L and 0.8 L were allocated for each culture and biogas (headspace), respectively. Cellulose (20 g/L, Sigma cat# C8002) was added and mixed into three of the replicate cultures, while the remaining two bioreactors were not supplemented with any substrate. Each culture was maintained for a period of 14 days, at a temperature of 38 °C, with agitation at 150 rpm using a Rushton-style impeller fixed to a stirring shaft built into the bioreactor. Each bioreactor was equipped with a flexible plastic tube to allow for the release of excess biogas and prevent pressure build-up. Samples for analysis (approximately 15 mL/sample) were collected from the rumen fluid inoculum immediately prior to culture setup (day 0 or D0), after 7 days in culture (day 7 or D7), and after 14 days in culture (day 14 or D14). Samples were immediately frozen after collection, then stored at −20 °C until processed. A total of three experiments were performed, each using a different fistulated animal as rumen fluid donor, which were designated as cow A, cow B and cow C, respectively.

### 2.2. Microbial DNA Extraction and PCR Amplification of the 16S rRNA Gene

Microbial DNA was isolated from rumen and culture samples using the repeated bead beating plus column method, as previously described [16]. Briefly, 250 µL of sample were lysed by bead beating with 0.4 g of zirconium beads at 3000 rpm (3 min) in lysis buffer (0.5 M NaCl, 50 mM Tris.HCl, 50 mM EDTA, 4% SDS), followed by heat treatment at 70 °C (15 min). Lysate was recovered by centrifugation (14,000× *g* or greater, 5 min, 4 °C). SDS and impurities were removed by ammonium acetate precipitation (10 M, 20% volume). DNA was recovered from the lysate using isopropanol precipitation, then further purified using a commercial kit following the manufacturer’s specifications (QIAamp DNA Stool Kit, QIAGEN, Hilden, Germany). The V1–V3 region of bacterial 16S rRNA gene sequences was PCR-amplified using the 27F forward [17] and 519R reverse [18] primer pair. PCR reactions were performed with the Phusion Taq DNA polymerase (Thermo Scientific) under the following conditions: hot start (4 min, 98 °C), followed by 35 cycles of denaturation (10 s, 98 °C), annealing (30 s, 50 °C) and extension (30 s, 72 °C), then ending with a final extension period (10 min, 72 °C). PCR products were separated by agarose gel electrophoresis, and amplicons of the expected size (~500 bp) were excised for gel purification using the QiaexII Gel extraction kit (QIAGEN). For each sample, approximately 400 ng of amplified DNA were submitted to Molecular Research DNA (MRDNA, Shallowater, TX, USA) for sequencing with the MiSeq 2x300 platform (Illumina, San Diego, CA, USA) to generate overlapping paired end reads. 

### 2.3. Computational Analysis of PCR-Generated 16S rRNA Amplicon Sequences 

Unless specified otherwise, the following steps were performed using custom written Perl scripts (available upon request). Raw bacterial 16S rRNA gene V1–V3 amplicon sequences were provided by Molecular Research DNA as assembled contigs from overlapping MiSeq 2 × 300 paired-end reads from the same flow cell clusters. Reads were then selected to meet the following criteria: presence of both intact 27F (forward) and 519R (reverse) primer nucleotide sequences, length between 400 and 580 nt, and a minimal quality threshold of no more than 1% of nucleotides with a Phred quality score lower than 15. The average Phred quality score of reads selected for analysis per sample ranged from 37.3 to 37.6 (Supplementary Table S1).

Following quality screens, sequence reads were aligned, then clustered into Operational Taxonomic Units (OTUs) at a genetic distance cutoff of 5% sequence dissimilarity. We have previously assessed [19] that a 5% dissimilarity cutoff for 16S rRNA is more representative of the genetic variation in 16S rRNA gene sequences for the V1–V3 hypervariable regions, when considering the meta-analysis by Kim et al. [20] as well as the level of nucleotide sequence dissimilarity among the bacterial species of well-populated genera such as *Clostridium*, *Prevotella*, or *Streptococcus*. OTUs were screened for DNA sequence artifacts using the following methods. Chimeric sequences were first identified with the chimera.uchime and chimera.slayer commands from the MOTHUR (v.1.36.1, University of Michigan, Ann Arbor, MI, USA) open source software package [21]. Secondly, the integrity of the 5′ and 3′ ends of OTUs was evaluated using a database alignment search-based approach; when compared to their closest match of equal or longer sequence length from the NCBI nt database, as determined by BLASTN (2.5.0) [22], OTUs with more than five nucleotides missing from the 5′ or 3′ end of their respective alignments were discarded as artifacts. Single read OTUs were subjected to an additional screen, where only sequences that had a perfect or near perfect match to a sequence in the NCBI nt database were kept for analysis, that is, that the alignment had to span the entire sequence of the OTU, and a maximum of 1% of dissimilar nucleotides was tolerated.

After removal of sequence chimeras and artifacts, the bacterial composition of each sample was determined by calculating the relative abundance of valid OTUs. This was defined as the number of sequence reads assigned to an OTU in a given sample, divided by the number of total reads in that sample. Rarefaction analysis was performed using MOTHUR (v.1.36.1), and results are presented in Figure S1 [21]. Taxonomic assignment of valid OTUs was determined using a combination of RDP Classifier [23] and BLAST [22]. The List of Prokaryotic Names with Standing in Nomenclature (LPSN) was also consulted for information on valid species belonging to taxa of interest [24,25].

### 2.4. Accession Numbers for Next Generation Sequencing Data

Raw sequence data is available from the NCBI Sequence Read Archive. Accession numbers are provided in Supplementary Table S1.

### 2.5. Short Chain Fatty Acids (SCFA) Analysis

SCFAs are products of interest from the digestion of plant structural polysaccharide by ruminal microorganisms, because they are absorbed and utilized by the host. For rumen or batch culture samples collected for SCFA composition analysis, metaphosphoric acid (25%) was added at a 1:4 ratio before storage at −20 °C. Samples were then thawed and centrifuged at 16,000× *g* (1 min) to remove particulates. SCFA were separated by GLC (Trace 1310, Thermo Scientific, Bellefonte, PA, USA) on a 0.25 mm i.d. × 30 m capillary column with 0.25 μm film thickness (Nukol, Thermo Scientific, Bellefonte, PA, USA). The injector port temperature was 200 °C, with a split ratio of 100:1, and a column flow of He at a rate of 0.8 mL/min. After starting at 140 °C for a duration of 9.5 min, the oven temperature was increased at a rate of 20 °C/min until it reached 200 °C, at which point it was maintained for 1 min. A mixture of SCFA (Supelco Volatile Free Fatty Acid Mix 46975, Supelco Inc., Bellefonte, PA, USA) was first analyzed for identification of short-chain fatty acids in the supernatant, with 2-ethylbutyric acid serving as an internal standard. Detection was completed using a flame-ionization detector with a temperature of 250 °C.

## 3. Results

### 3.1. Bacterial Community Structure of Rumen Samples Prior to Selection with Cellulose

The composition of bacterial communities in rumen samples collected from three beef cows maintained on a forage diet was determined prior to selection with cellulose (Table 1). Taxonomic analysis revealed that the phylum Bacteroidetes was the most abundant in each of the samples (47.3–60.5% of reads per sample), with Prevotellaceae identified as the most highly represented family (19.2–40.5% of reads per sample). Firmicutes was the second most abundant phylum, ranging from 21.7 to 32.6%. Sequences assigned to Clostridiales, mainly Ruminococcaceae, Lachnospiraceae and unclassified Clostridiales, represented 89.4–95.5% of Firmicutes sequences.

**Figure 1:**
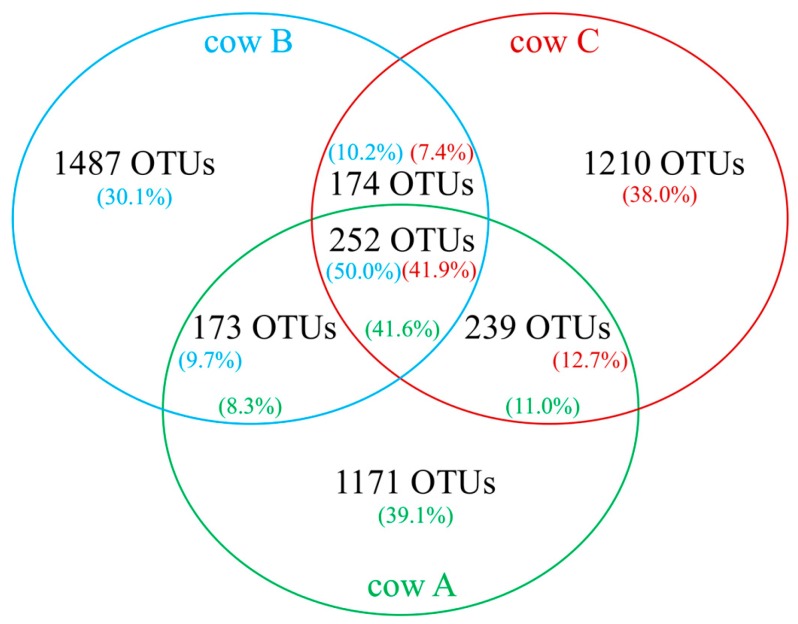
Venn diagram showing the number of shared rumen bacterial OTUs amongst three beef cows maintained under a forage diet. Also shown is the proportion of sequence reads from each rumen sample that were assigned to shared OTUs.

Further analyses revealed that 252 OTUs were shared amongst the three rumen samples, which represented 41.6–50.0% of reads within corresponding samples (Figure 1). Individually, however, shared OTUs varied in abundance and prominence amongst different rumen samples (Table 2). Indeed, only OTU SD_Bt-00012 (genus *Fibrobacter*, phylum Fibrobacteres) was found at an abundance greater than 1% in all samples, with its highest representation in cow B (5.6%). When samples were compared as pairs, other OTUs displayed abundance values within the same range, which included OTUs SD_Bt-00007 (4.8–5.0%), SD_Bt-00030 (1.4%), SD_Bt-00051 (1.2–1.3%) and SD_Bt-00087 (1.0–1.4%). Amongst these OTUs, SD_Bt-00012 and SD_Bt-00087 were the most likely to correspond to characterized species, as they displayed a sequence identity of 98.8% or greater to their respective closest relative (Table 2). 

### 3.2. Effect of Cellulose Selection on the Composition of Rumen Bacterial Populations

**Figure 2:**
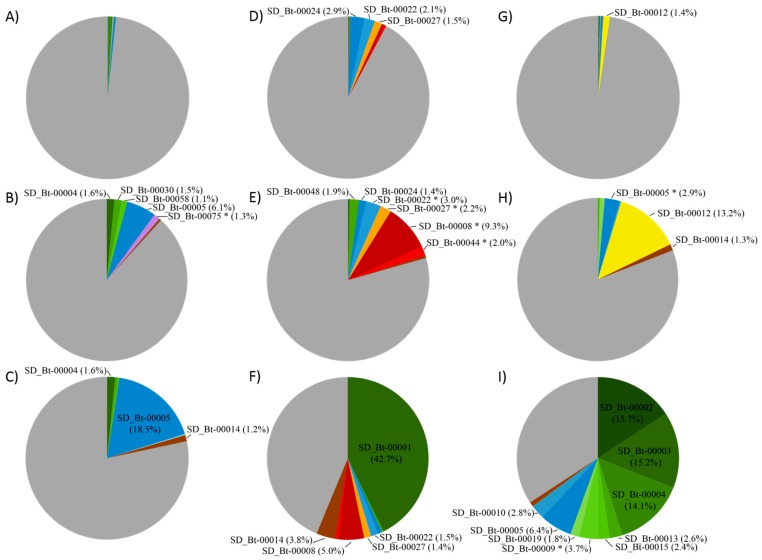
Composition of bacterial consortia in response to cellulose, presented as relative abundance (%). OTUs enriched in the presence of cellulose with a relative abundance of at least 1% from at least one time point are indicated. OTUs found to be statistically higher in cellulose supplemented cultures compared to their corresponding non supplemented controls are indicated (*). Cow A: (**A**) rumen sample (D0); (**B**) D7 cultures and (**C**) D14 cultures. Cow B: (**D**) rumen sample (D0); (**E**) D7 cultures and (**F**) D14 cultures. Cow C: (**G**) rumen sample (D0); (**H**) D7 cultures and (**I**) D14 cultures. OTU abundances at D7 and D14 are the average of three replicate cultures at the indicated time point. Distinct OTUs assigned to the same phylum are shown in shades of the same color: Bacteroidetes (blue), Firmicutes (green), Candidatus Saccharibacteria (red), Proteobacteria (purple), Fibrobacteres (yellow), Planctomycetes (orange), and unclassified Bacteria (brown). The remaining OTUs from each sample were grouped in the same category (grey). Relative abundances of OTUs in Control cultures are presented in Supplementary Tables S2–S4.

To identify cellulose-utilizing OTUs, a batch culture approach was used. From the rumen bacterial communities of cow A, OTU SD_Bt-00005 showed the greatest enrichment in response to cellulose, increasing from 0.4% to 6.1% and 18.5% in D7 and D14 cultures (Figure 2), respectively. Compared to control cultures (Supplemental Table S2), an enrichment of 33.0 and 114.1 fold on D7 and D14, respectively, was observed. While their relative abundance on D14 was lower than SD_Bt-00005, SD_Bt-00004 (1.6%) and SD_Bt-00014 (1.2%) also showed similar levels of enrichment in cellulose-supplemented cultures compared to controls, with increases of 17.8–23.0 fold on D7 and 74.0–95.9 fold on D14. Together, enriched OTUs from cow A represented 12.0% and 22.4% of bacteria in D7 and D14 cellulose-supplemented cultures, while their combined abundance in their respective control cultures was 0.8% and 0.7% (Supplemental Table S2).

OTU SD_Bt-00001, from cow B, showed the highest overall level of enrichment from any experiment from this study, reaching 42.7% on D14 supplemented cultures, an increase of 152.8 fold compared to its sample of origin (Figure 2 and Supplemental Table S3). Intriguingly, this increase in relative abundance took place during the second week, as its levels in D7 cultures were much lower (0.3%). A very distinct feature of cellulose-supplemented cultures from this sample was the enrichment of two OTUs, SD_Bt-00008 and SD_Bt-00044, assigned to the candidate lineage Saccharibacteria [26], formerly proposed as the TM7 division [27]. These OTUs were found in greatest abundance in D7 cultures, with enrichments of 27.5- and 12.6-fold, respectively, compared to control cultures (Supplemental Table S3). OTU SD_Bt-00014, which could not be assigned to either a valid or candidate phylum, increased from 0.01% in the original rumen sample to 3.8% after 14 days in the presence of cellulose. Together, OTUs enriched from cow B represented 20.6% and 56.3% of 16S rRNA sequences in D7 and D14 cultures, respectively, compared to 1.9% and 1.2% in D7 and D14 controls, respectively.

Cellulose enrichment from cow C resulted in the most complex consortial composition pattern observed in this study (Table 3). OTU SD_Bt-00012, likely a strain of *Fibrobacter succinogenes* (98.8% sequence identity) increased from 1.4% in the original sample to 13.2% by D7 in supplemented cultures, but then was greatly reduced in D14 cultures (0.1%). Three OTUs (SD_Bt-00002, SD_Bt-00003, and SD_Bt-00004) showed the highest response to culturing in the presence of cellulose, each with abundances ranging from 14.1% to 15.7% in D14 cultures, while only displaying limited increases on D7 (0.2–0.6%). Six other OTUs (SD_Bt-00005, SD_Bt-00009, SD_Bt-00010, SD_Bt-00013, SD_Bt-00015, and SD_Bt-00019) were also found in greater abundance on D14 (1.8–6.4%) compared to their sample of origin (0–0.4%) or control cultures at the same time point (0.01–0.1%). Together, enriched OTUs represented 19.2% of sequences on D7 and 65.8% on D14, compared to 1.2% and 1.4% in their respective controls.

### 3.3. SCFA Profile Analysis from Cellulose-Utilizing Consortia

As the main function of rumen microbial communities is the digestion of plant structural polysaccharides into SCFAs, mainly acetate, propionate, and butyrate, we aimed to gain further insights into the microbial ecology of cellulose-mediated consortium assembly from rumen bacterial community through investigation of SCFA profiles. 

Cultures derived from cow C were selected as they had the most complex cellulose-enriched consortium obtained in this study. Compared to their rumen sample of origin, both control and cellulose-supplemented cultures showed increased SCFA concentrations after seven days (Table 4). However, only isovalerate was found to be significantly different between control and cellulose supplemented cultures at this time point. At day 14, none of the SCFAs analyzed were found to be statistically different.

## 4. Discussion

Microorganisms that populate the rumen compartment of the gastro-intestinal tract are responsible for digesting the different components of feed, producing end products in the form of SCFAs that can be absorbed and metabolized by their ruminant host [28]. Due to many factors, the vast complexity of this ecosystem has yet to be fully elucidated. Indeed, the wide array of chemical ingredients that compose ruminant diets, the molecular interactions between host and symbionts, as well as the high degree of specialization and functional overlap amongst anaerobic microorganisms represent some of the major determinants or modulators of rumen microbial composition [1,15]. 

Since cellulose is the most abundant component of forage-based diets, there is considerable interest in further elucidating the mechanisms responsible for its digestion in ruminants. As the necessary enzymes are not encoded in the genome of their host, ruminal microbial symbionts are essential for this process [29]. While a number of cellulose-utilizing rumen bacterial species have been isolated over the years, metagenomics studies have indicated that the vast majority remain to be characterized [10,11,14,30]. In an effort to gain further insight, we have described in this report the composition of cellulose-utilizing bacterial consortia that were enriched from the respective rumen of three beef cows maintained on a forage diet. Unexpectedly, while these rumen bacterial communities were found to share 252 OTUs, which together accounted for 41.6–50.0% of their respective sequence dataset, only one OTU (SD_Bt-00014) was enriched in cultures from all animals. SD_Bt-00014 was found in low abundance in all rumen samples (0.01–0.05%), as well as in non-supplemented controls (0.02–0.05%). While it could not be assigned to any currently known phyla, SD_Bt-00014 has previously been identified in the rumen contents of Hereford-Aberdeen Angus crossbred steers raised under feedlot conditions on a finishing diet composed of barley grain and barley silage [31]. OTUs SD_Bt-00004 and SD_Bt-00005 were enriched from two of the original samples (cow A and cow C). While SD_Bt-00005 was one of the 252 core OTUs, SD_Bt-00004 was found in cow A and cow C, but not in cow B. Based on their respective 16S rRNA gene sequences, SD_Bt-00004 and SD_Bt-00005 were predicted to likely correspond to uncultured species of Ruminococcaceae and Prevotellaceae, respectively. Apart from these OTUs, each set of replicate cellulose-supplemented cultures originating from a given rumen environment was found to have a distinct bacterial composition compared to cultures generated from other animals. While future research will be necessary to elucidate the mechanisms involved, these results suggest that extensive overlap in bacterial composition amongst rumen environments adapted to a forage diet is not sufficient to predetermine the nature of the species that will assemble into consortia in response to cellulose. 

Amongst the various cellulose enriched OTUs described in this report, seven were found in distinctively higher abundance in supplemented cultures. Two of these OTUs were matched to well-characterized cellulose-utilizing species. Indeed, based on a genetic distance cutoff of 5%, SD_Bt-00001 and SD_Bt-00012 would have represented strains of *R. flavefaciens* and *F. succinogenes*, respectively. *R. flavefaciens* can metabolize cellulose through the expression of cellulosomes [32], as well as other carbohydrate-utilizing enzymes, such as endocellulases, glucanases, exoglucanases, and cellodextrinases [33,34,35,36,37,38]. In addition to its versatility in utilization of plant cell wall polysaccharides, it can metabolize crystalline cellulose at a rate higher than other fibrolytic rumen bacteria, suggesting that it likely thrives in fiber-rich environments [39,40]. While OTUs related to *R. flavefaciens* have commonly been reported from ruminal diversity studies, they are typically found in relatively low abundance. For instance, two distinct strains of *R. flavefaciens*, which represented 0.06% to 0.08% of the total reads on a scaled average among seven studies, were included as core rumen bacteria by Creevy et al. [9]. While SD_Bt-00001 was only detected in cow B (0.3%), another predicted strain of *R. flavefaciens*, OTU SD_Bt-00058, was present in all three rumen samples (0.2% in cow A, 0.07% in cow B and 0.6% in cow C). However, it was only slightly enriched in cellulose-supplemented cultures from cow A on D7 (Supplemental Table S2). *F. succinogenes* is also well known to grow in vitro on medium supplemented with purified cellulose [41], but the mechanisms by which it is able to breakdown its substrate still remain to be determined. Indeed, its genome does not appear to possess open reading frames for components of cellulosomes or other known polysaccharide utilizing complexes, but rather encodes for other types of enzymes and accessory proteins that can perform the same function [42].

The remaining OTUs found to be highly enriched in cellulose-supplemented cultures did not correspond to currently defined bacterial species. SD_Bt-00008 was assigned to the candidate phylum Saccharibacteria, a phylogenetic lineage of uncultured bacteria that was defined genetically as a result of diversity and metagenomic analyses from a wide range of habitats, such as wastewater, soil and animal gastrointestinal tract [26,27]. SD_Bt-00002, predicted to belong to the genus *Ethanoligenens* of the family Ruminococcaceae, was identified from the rumen of cow C. Originally isolated from the anaerobic sludge of molasses wastewater, *E. harbinense* is thus far the only cultured representative of its genus. It is capable of fermenting various mono-, di- and oligosaccharides, including d-glucose and cellobiose, into ethanol, acetate, hydrogen, and carbon dioxide [43]. Based on these metabolic activities, co-enrichment of an uncharacterized species of *Ethanoligenens* suggests that it may benefit from the cellulose metabolizing activity of other rumen bacteria, such as perhaps SD_Bt-00003 and/or SD_Bt-00004, that were also enriched from the same animal. These results would be consistent with reports from other research groups. Indeed, *E. harbinense* was found to be among the main species enriched in the second generation of a repeated-batch culture of ruminal fluid supplemented with napiergrass [44], and an *Ethanoligenens*-related OTU was recently identified from rumen fluid supplemented with wheat straw [45]. While SD_Bt-00002 and other uncharacterized rumen species of *Ethanoligenens* may be capable of metabolizing cellulose, future investigations will be required to determine their metabolic activities in the gastro-intestinal tract of ruminants.

The remaining two enriched OTUs affiliated to Ruminococcaceae, SD_Bt-00003 and SD_Bt-00004, were designated as unclassified members of this family of Firmicutes. Based on their limited sequence identity to their closest valid relative (Table 3), they may have represented novel genera of Ruminococcaceae. No closely related sequences to these OTUs could be identified from the NCBI nt database (data not shown). In their meta-analysis, Kim et al. [20] found that unclassified Ruminococcaceae represented 8.7% of publicly available full length bacterial rumen 16S rRNA gene sequences, a higher proportion than the remaining members of this family that could be assigned to a known genus (4.8%). Evidence supporting the involvement of this group in fiber degradation has been provided by other research efforts. Indeed, unclassified Ruminococcaceae were found to be in similar abundance (5.6%) in the rumen of 2-year old Israeli Holsteins fed a 70% concentrate: 30% roughage diet [46], as well as to increase within 8 hours of in situ incubation with fresh perennial ryegrass [47]. Furthermore, an unclassified Ruminococcaceae OTU was one of the most abundant groups of bacteria in the rumen of cows consuming a forage diet [48]. 

Members of the genus *Prevotella* are considered the most predominant group of ruminal bacteria [20,49], which is also supported by data from this study. Several cellulolytic species of this genus have been previously described, including *P. bryantii*, *P. ruminicola*, and *P. albensis*, and they are regularly identified in rumen diversity studies [49,50]. The importance of *Prevotella* in plant fiber digestion in the rumen is supported by the number of enzymes, such as endocellulases, endogluconases, and exogluconases, that have been identified in members of this genus [51,52,53]. Further support for their contribution in fiber degradation was provided by a metagenomics study showing that at least 36% of the glycoside hydrolases and other carbohydrate-metabolizing enzymes from animals fed finger millet straw were affiliated to *Prevotella* [54]. However, while Prevotellaceae was the most abundant group in the rumen samples analyzed in this study, their relative abundance decreased after 7 days when cellulose was the only exogenous substrate provided, and only one OTU (SD_Bt-00005) affiliated to this group was enriched in the presence of cellulose. Since other *Prevotella* species are involved in the breakdown of starch or proteins rather than cellulose [1,55], perhaps members of this group were primarily involved in metabolizing other components of the diet fed to the animals sampled in this study.

Together, these results highlight cellulose utilization as a metabolic trait shared across vast phylogenetic distances in the rumen, further supporting this environment as conducive to the selection of a broad range of microbial adaptations for the digestion of plant structural polysaccharides. The results from the current study add to metagenomic analyses that previously reported a high level of functional redundancy for cellulose utilization amongst rumen microorganisms, as this substrate appears to support a very diverse array of low abundance microorganisms [10,11,12,13,14,15]. While the mechanisms responsible for such wide diversity remain to be determined, each cellulose-utilizing species may express distinct properties or activities that contribute to other ruminal functions. Such features could explain how only a limited number of common OTUs were obtained after selection on cellulose from rumen microbial ecosystems that shared core OTUs representing almost half of their respective bacterial communities.

## Figures and Tables

**Table 1:** Taxonomic profile of rumen bacteria from three beef cows on a forage-based diet. For each sample, relative abundances (%) are shown for all Operational Taxonomic Units (OTUs) as well as for the 252 OTUs shared among animals. Phyla are highlighted in bold, with most highly represented lower taxa shown for Bacteroidetes and Firmicutes.

Taxonomic Affiliation	All OTUs			Shared OTUs		
	Cow A	Cow B	Cow C	Cow A	Cow B	Cow C
Bacteroidetes	48.0	47.3	60.5	18.0	22.4	25.9
Prevotellaceae	27.4	19.2	40.5	11.3	10.6	18.3
Porphyromonadaceae	0.5	7.4	0.5	0.3	3.7	0.2
unc. Bacteroidales	14.2	14.2	14.7	3.6	5.2	5.7
other Bacteroidetes	5.9	6.5	4.8	2.7	3.0	1.6
Firmicutes	32.6	21.7	31.4	11.3	10.0	11.5
Ruminococcaceae	10.3	5.9	12.4	3.6	2.8	4.3
Lachnospiraceae	10.5	8.3	9.2	4.4	3.5	3.7
unc. Clostridiales	10.0	5.2	8.4	2.9	2.5	3.1
other Firmicutes	1.8	2.3	1.4	0.4	1.2	0.4
Fibrobacteres	2.6	8.2	2.4	2.5	7.8	1.9
Plantomycetes	0.4	3.9	0.09	0	0	0
Cand. Saccha.	0.2	1.0	0.1	0.08	0.6	0.09
Proteobacteria	1.1	0.03	0.4	0.7	1.7	0.1
other phyla	5.1	7.1	1.8	3.4	1.0	1.1
unc. Bacteria	9.9	10.8	3.2	5.7	6.4	1.4
Total	100	100	100	41.6	50.0	41.9

Abbreviations: Cand. Saccha.: Canditatus Saccharibacteria; unc.: unclassified.

**Table 2:** Most abundant ^a^ rumen bacterial OTUs from three beef cows maintained on a forage diet. Representation is shown for each animal (cow A, cow B, cow C) as relative abundance, with corresponding taxonomic assignments and closest taxon also presented.

OTU	A	B	C	Taxonomic Affiliation ^b^	Closest Valid Taxon (id% ^c^)
SD_Bt-00007	4.8	5.0	0.9	unc. Bacteria	*Clostridium hveragerdense* (80.8)
SD_Bt-00012	1.6	5.6	1.4	*Fibrobacter*	*F. succinogenes* (98.8)
SD_Bt-00022	0	2.1	0.1	*Prevotella*	*P. ruminicola* (92.2)
SD_Bt-00024	0.2	2.9	0.1	unc. Porphyromonadaceae	*Paludibacter jiangxiensis* (83.6)
SD_Bt-00027	0	1.5	0	unc. Planctomycetaceae	*Rhodopirellula baltica* (80.6)
SD_Bt-00030	0.9	1.4	1.4	*Prevotella*	*P. ruminicola* (91.3)
SD_Bt-00033	0.2	1.3	0.1	unc. Bacteria	*Victivallis vadensis* (82.8)
SD_Bt-00039	0.1	1.5	0	unc. Planctomycetaceae	*Rhodopirellula lusitana* (83.6)
SD_Bt-00040	0.6	1.0	0	unc. Bacteroidales	*Butyricimonas faecihominis* (82.4)
SD_Bt-00051	1.2	1.3	0.8	unc. Bacteroidetes	*Odoribacter splanchnicus* (82.5)
SD_Bt-00057	2.2	0.2	0.6	SR1	*Leifsonia shinshuensis* (78.1)
SD_Bt-00062	0.1	0.8	2.5	unc. Bacteroidales	*Butyricimonas virosa* (83.1)
SD_Bt-00074	0.5	0.3	1.9	*Prevotella*	*P. ruminicola* (93.2)
SD_Bt-00084	1.3	0.3	0.5	*Prevotella*	*P. ruminicola* (90.4)
SD_Bt-00087	1.4	1.0	0.7	*Pseudobutyrivibrio*	*Ps. ruminis* (99.0)
SD_Bt-00108	0.1	1.1	0	*Succinivibrio*	*S. dextrinosolvens* (99.2)
SD_Bt-00112	0.4	1.0	0	unc. Bacteroidales	*P. conceptionensis* (83.4)
SD_Bt-00118	0.8	0	1.3	*Prevotella*	*P. ruminicola* (91.7)
SD_Bt-00119	0.4	0.1	1.1	*Prevotella*	*P. ruminicola* (91.0)
SD_Bt-00142	0.5	1.2	0	*Fibrobacter*	*F. succinogenes* (95.6)
SD_Bt-00160	0.5	0.2	1.0	unc. Bacteroidales	*P. ruminicola* (88.5)
SD_Bt-10002	0	1.0	0	unc. Bacteroidales	*Butyricimonas faecihominis* (83.4)
SD_Bt-10017	0	1.0	0.1	*Prevotella*	*P. ruminicola* (88.4)
SD_Bt-10023	0	1.4	0	*Kerstersia*	*Devosia riboflavina* (84.0)
Total	17.8	33.2	14.5		

^a^ OTUs representing at least 1% of sequence reads in at least one of the rumen samples; ^b^ As determined by RDP classifier [23], at an 80% bootstrap cutoff; ^c^ Search result and nucleotide sequence identity as determined by BLAST [22]. Abbreviation: unc.: unclassified.

**Table 3:** Taxonomic affiliation of the most abundant ^a^ OTUs from selection with cellulose.

OTUs	Taxonomic Affiliation ^b^	Closest Valid Taxon (id% ^c^)
SD_Bt-00001	*Ruminococcus*	*Ruminococcus flavefaciens* (95.2)
SD_Bt-00002	*Ethanoligenens*	*Clostridium cellulosi* (93.6)
SD_Bt-00003	unc. Ruminococcaceae	*Anaerotruncus colihominis* (89.3)
SD_Bt-00004	unc. Ruminococcaceae	*Clostridium cellulosi* (92.5)
SD_Bt-00005	*Prevotella*	*Prevotella bivia* (90.2)
SD_Bt-00008	*Saccharibacteria gen. inc. sed.*	*Cand. Saccharibacteria* ^d^ (89.9)
SD_Bt-00009	unc. Lachnospiraceae	*Butyrivibrio fibrisolvens* (91.3)
SD_Bt-00010	*Prevotella*	*Prevotella albensis* (97.7)
SD_Bt-00012	*Fibrobacter*	*Fibrobacter succinogenes* (98.8)
SD_Bt-00013	unc. Ruminococcaceae	*Anaerotruncus colihominis* (89.3)
SD_Bt-00014	unc. Bacteria	*Solobacterium moorei* (88.0)
SD_Bt-00015	unc. Ruminococcaceae	*Clostridium cellulosi* (91.5)
SD_Bt-00019	unc. Ruminococcaceae	*Clostridium cellulosi* (91.2)
SD_Bt-00022	*Prevotella*	*Prevotella ruminicola* (92.2)
SD_Bt-00024	unc. Porphyromonadaceae	*Paludibacter jiangxiensis* (83.6)
SD_Bt-00027	unc. Planctomycetaceae	*Rhodopirellula baltica* (80.6)
SD_Bt-00030	*Prevotella*	*Prevotella ruminicola* (91.3)
SD_Bt-00044	*Saccharibacteria gen. inc. sed.*	*Cand. Saccharibacteria* ^d^ (90.6)
SD_Bt-00048	unc. Ruminococcaceae	*Saccharofermentans acetigenes* (87.8)
SD_Bt-00058	*Ruminococcus*	*Ruminococcus flavefaciens* (96.4)
SD_Bt-00075	unc. Proteobacteria	*Devosia geojensis* (84.4)

^a^ OTUs representing at least 1% of sequence reads in cellulose-enriched cultures; ^b^ As determined by RDP classifier [23], at an 80% bootstrap cutoff; ^c^ Nucleotide sequence identity as determined by BLAST [22]; ^d^ Genome CP011211.1. Abbreviations: *Cand.*: *Canditatus*; *gen. inc. sed.*: *genera incertae sedis*; unc.: unclassified.

**Table 4:** Short Chain Fatty Acids (SCFA) profile (mM) from the rumen sample (D0) and cultures derived from cow C.

	D0	Con ^a^ D7	Cell ^b^ D7	Con ^a^ D14	Cell ^b^ D14
Acetate	50.18	90.55 ± 11.06	95.48 ± 7.57	76.50 ± 9.92	90.73 ± 10.90
Propionate	12.46	20.35 ± 2.72	23.01 ± 0.78	18.75 ± 2.47	22.13 ± 2.57
Butyrate	8.18	11.22 ± 1.03	12.20 ±0.33	10.40 ± 0.75	12.45 ± 0.94
Iso-butyrate	0.79	1.77 ± 0.22	1.51 ± 0.06	1.73 ± 0.15	1.55 ± 0.12
Valerate	0.98	1.82 ± 0.20	1.86 ± 0.05	1.73 ± 0.11	2.19 ± 0.25
Iso-valerate	1.13	3.12 * ± 0.33	2.47 * ± 0.09	3.10 ± 0.23	2.57 ± 0.19

* Control—Cellulose pairs for the same time point that are statistically different (unpaired *t*-test, *p* < 0.05); ^a^ Control: average and standard deviation of duplicate cultures; ^b^ Cellulose supplementation: average and standard deviation of triplicate cultures.

## Supplementary Materials

The following are available online at http://www.mdpi.com/2076-2607/6/1/17/s1. Figure S1: Rarefaction analysis of sequence read coverage for each sample, Table S1: Read numbers, average quality scores, and SRA accession numbers for 16S rRNA reads used in analysis of each sample, Tables S2–S4: Relative abundance of each OTU identified from rumen samples and rumen-derived cultures.

Supplementary File 1
