# Supplementary material for: Identification of Uncultured Bacterial Species from Firmicutes, Bacteroidetes and CANDIDATUS Saccharibacteria as Candidate Cellulose Utilizers from the Rumen of Beef Cows
Source: Microorganisms. 2018 Feb 24;6(1):17. doi: 10.3390/microorganisms6010017 (PMC5874631; doi:10.3390/microorganisms6010017)
Supplement: Supplementary File 1 [file microorganisms-06-00017-s001.zip › Opdahl et al supplemental Revised/Opdahl et al Supp Table 1.docx]

**Supplementary Table S1.** Number of high-quality (Q15) and non-chimeric 16S rRNA reads used to determine the bacterial composition of samples from rumen (inoculum - D0), Controls (D7, D14) and Cellulose-enriched cultures (D7, D14) from each animal.

|  | **Sample** | **Q15^a^ reads** | **Phred^b^ / read** | **Non chimeric^c^ reads** | **SRA accession numbers^d^** |
| --- | --- | --- | --- | --- | --- |
| **Cow A** | D0 Inoculum | 15905 | 37.5 | 3,906 | SRR6211598 |
| **Cow A** | D7 Control-1 | 19646 | 37.6 | 5,832 | SRR6211597 |
| **Cow A** | D7 Control-2 | 21237 | 37.5 | 8,449 | SRR6211595 |
| **Cow A** | D7 Cellulose-1 | 23819 | 37.5 | 9,788 | SRR6211594 |
| **Cow A** | D7 Cellulose-2 | 23382 | 37.5 | 9,085 | SRR6211602 |
| **Cow A** | D7 Cellulose-3 | 22155 | 37.5 | 9,729 | SRR6211601 |
| **Cow A** | D14 Control-1 | 11742 | 37.5 | 2,970 | SRR6211600 |
| **Cow A** | D14 Control-2 | 11220 | 37.5 | 2,452 | SRR6211599 |
| **Cow A** | D14 Cellulose-1 | 15866 | 37.5 | 7,412 | SRR6211593 |
| **Cow A** | D14 Cellulose-2 | 12381 | 37.4 | 6,864 | SRR6211596 |
| **Cow A** | D14 Cellulose-3 | 21261 | 37.5 | 7,287 | SRR6211606 |
|  |  |  |  |  |  |
| **Cow B** | D0 Inoculum | 21856 | 37.4 | 13,612 | SRR6211605 |
| **Cow B** | D7 Control-1 | 15001 | 37.4 | 7,720 | SRR6211607 |
| **Cow B** | D7 Control-2 | 27543 | 37.3 | 19,288 | SRR6211610 |
| **Cow B** | D7 Cellulose-1 | 20354 | 37.3 | 14,912 | SRR6211609 |
| **Cow B** | D7 Cellulose-2 | 21074 | 37.4 | 12,380 | SRR6211608 |
| **Cow B** | D7 Cellulose-3 | 13303 | 37.4 | 6,521 | SRR6211612 |
| **Cow B** | D14 Control-1 | 20254 | 37.3 | 13,970 | SRR6211611 |
| **Cow B** | D14 Control-2 | 18081 | 37.4 | 10,364 | SRR6211604 |
| **Cow B** | D14 Cellulose-1 | 17044 | 37.4 | 12,890 | SRR6211603 |
| **Cow B** | D14 Cellulose-2 | 26968 | 37.4 | 18,591 | SRR6211622 |
| **Cow B** | D14 Cellulose-3 | 16656 | 37.3 | 8,678 | SRR6211623 |
|  |  |  |  |  |  |
| **Cow C** | D0 Inoculum | 13108 | 37.5 | 4,370 | SRR6211624 |
| **Cow C** | D7 Control-1 | 11758 | 37.5 | 3,644 | SRR6211625 |
| **Cow C** | D7 Control-2 | 9911 | 37.5 | 2,486 | SRR6211617 |
| **Cow C** | D7 Cellulose-1 | 15981 | 37.5 | 4,524 | SRR6211619 |
| **Cow C** | D7 Cellulose-2 | 17756 | 37.5 | 5,921 | SRR6211620 |
| **Cow C** | D7 Cellulose-3 | 14718 | 37.5 | 5,094 | SRR6211621 |
| **Cow C** | D14 Control-1 | 15013 | 37.5 | 5,167 | SRR6211615 |
| **Cow C** | D14 Control-2 | 6708 | 37.5 | 1,883 | SRR6211616 |
| **Cow C** | D14 Cellulose-1 | 64789 | 37.5 | 40,703 | SRR6211618 |
| **Cow C** | D14 Cellulose-2 | 51325 | 37.5 | 35,872 | SRR6211614 |
| **Cow C** | D14 Cellulose-3 | 61924 | 37.5 | 43,541 | SRR6211613 |

a. Number of reads for each sample that had both intact 27F (forward) and 519R (reverse) primer nucleotide sequences, length between 400 and 580 nt, and a minimal quality threshold of no more than 1% of nucleotides with a Phred quality score lower than 15.

b. For each sample, average Phred quality score per read (Q15 reads).

c. Number of reads used for bacterial composition analysis (i.e. after chimera check, 5’ and 3’ end quality check, as well as single-read OTU check).

d. Accession numbers for raw, unprocessed sequence reads with barcodes.
