# Supplementary material for: Identification of Uncultured Bacterial Species from Firmicutes, Bacteroidetes and CANDIDATUS Saccharibacteria as Candidate Cellulose Utilizers from the Rumen of Beef Cows
Source: Microorganisms. 2018 Feb 24;6(1):17. doi: 10.3390/microorganisms6010017 (PMC5874631; doi:10.3390/microorganisms6010017)
Supplement: Supplementary File 1 [file microorganisms-06-00017-s001.zip › Opdahl et al supplemental Revised/Rarefaction figure.pdf]

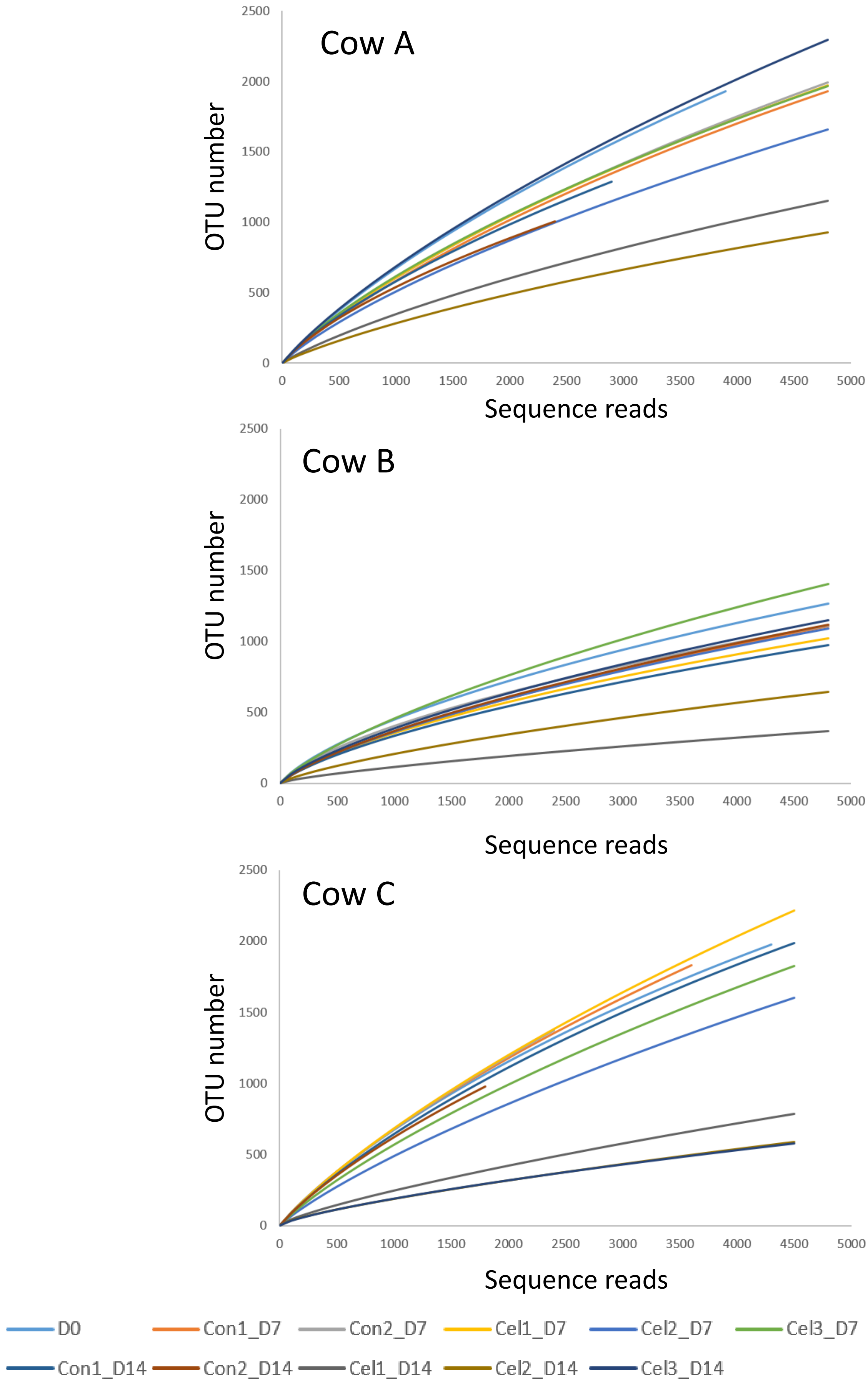

Figure S1. Rarefaction analysis of sequence read coverage for each sample
